# Diffused bladder wall calcification in a survivor with severe coronavirus disease 2019: A case report

**DOI:** 10.1097/MD.0000000000030314

**Published:** 2022-08-26

**Authors:** Pai-Yu Cheng, Yi-You Huang, Fu-Shan Jaw, Shiu-Dong Chung, Chung-You Tsai

**Affiliations:** a Department of Biomedical Engineering, College of Medicine and College of Engineering, National Taiwan University, Taiwan; b Divisions of Urology, Department of Surgery, Far Eastern Memorial Hospital, Taiwan; c Department of Nursing, College of Healthcare & Management, Asia Eastern University of Science and Technology, Taiwan; d General Education Center, Eastern University of Science and Technology, Taiwan; e Department of Electrical Engineering, Yuan Ze University, Taiwan.

**Keywords:** Case report [V03.100], COVID-19 [C01.925.705.500], Cystitis [C12.950.829.495], Systemic Inflammatory Response Syndrome [C23.550.470.790], Urolithiasis [C12.950.967]

## Abstract

Bladder calcification is a rare presentation that was first interpreted to be related to a urea-splitting bacterial infection. Aside from infection, other hypotheses such as schistosomiasis, tuberculosis, cancer, and cytokine-induced inflammatory processes have also been reported. Severe coronavirus disease 2019 (COVID-19) is known for its provoking cytokine storm and uninhibited systematic inflammation, and calcification over the coronary artery or lung has been reported as a long-term complication.

**Patient concerns:**

We presented a 68 years old man who had persistent lower urinary tract symptoms after recovery from severe COVID-19. No urea-splitting bacteria were identified from urine culture.

**Diagnosis:**

Cystoscopy examination revealed diffuse bladder mucosal and submucosa calcification.

**Interventions:**

Transurethral removal of the mucosal calcification with lithotripsy.

**Outcomes:**

The patient’s lower urinary tract symptoms improved, and stone analysis showed 98% calcium phosphate and 2% calcium oxalate. No newly formed calcifications were found at serial follow-up.

**Conclusion:**

Diffuse bladder calcification may be a urinary tract sequela of COVID-19 infection. Patients with de novo lower urinary tract symptoms after severe COVID-19 should be further investigated.

## 1. Introduction

Bladder calcification is a rare condition that describes calcification of either the bladder mucosal or submucosal layer. The causative pathogens are urease-producing bacteria, of which Corynebacterium urealyticum (CU) is commonly reported. Schistosomiasis, tuberculosis, cancer, and cytokine-induced inflammatory processes have also been reported to be associated with calcium deposition. As more coronavirus disease 2019 (Covid-19) studies have been published during the pandemic, growing evidence suggests a strong correlation between severe Covid-19 and a tremendously provoked cytokine storm. As more observational studies have found that patients with Covid-19 may report lower urinary tract symptoms due to viral cystitis, the manifestation of the urinary tract system in patients with Covid-19 is of great interest to the community. Here, we report a patient who had newly developed frequency and nocturia after severe Covid-19, and was diagnosed with bladder calcification.

## 2. Case report

**Figure 1.**
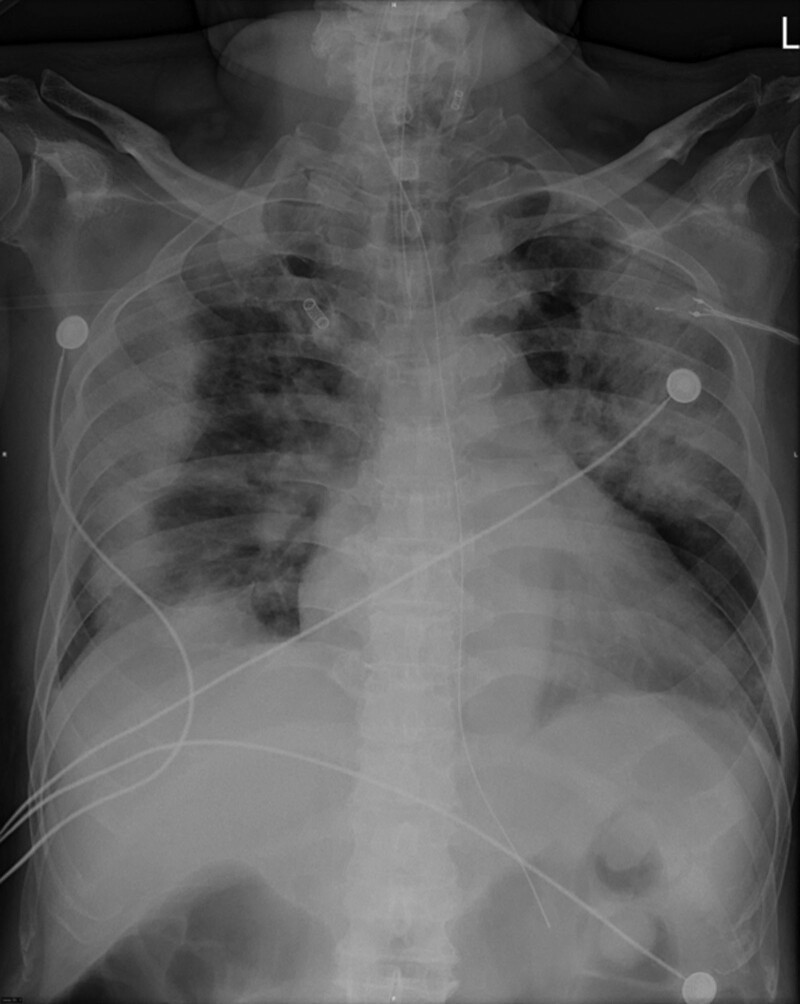
COVID-19 related acute respiratory distress syndrome.

A 68-year-old man presented to our urology clinic with urinary frequency and nocturia. He had hypertension and atrial fibrillation and was treated with amiodarone (200 mg QD). Two months prior, he had intermittent fever and productive cough and was diagnosed with severe Covid-19. No history of Covid-19 vaccination was contributed before. Rapid progression to acute respiratory distress syndrome was observed (Fig. 1), and he was intubated with mechanical ventilation support. Remdesivir, dexamethasone and clexane have been prescribed for the treatment of severe Covid-19. The Foley catheter was indwelled with frequent changes during intensive care period; urine culture for infection surveillance showed Candida metapsilosis and Candida parapsilosis. No Corynebacterium infection was identified throughout the hospital stay, and urine pH was normal. After one and a half months, he recovered from severe Covid-19 and was discharged without a ventilator.

**Figure 2.**
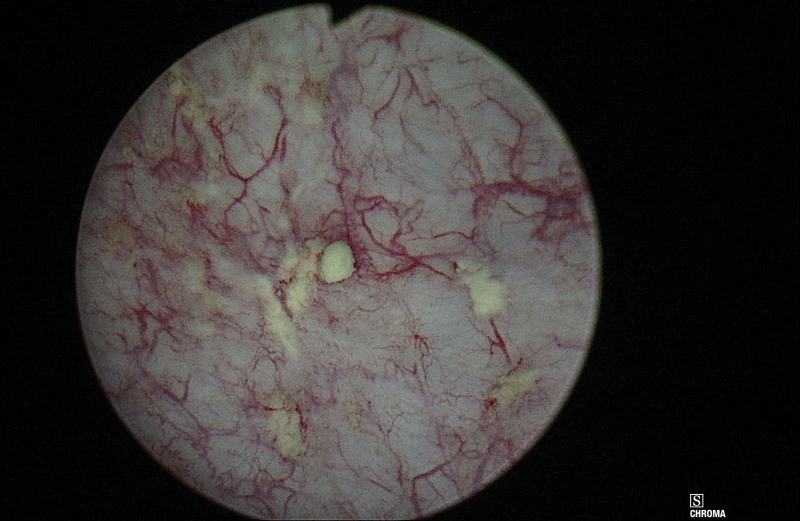
Bladder mucosal and submucosal calcification.

**Figure 3.**
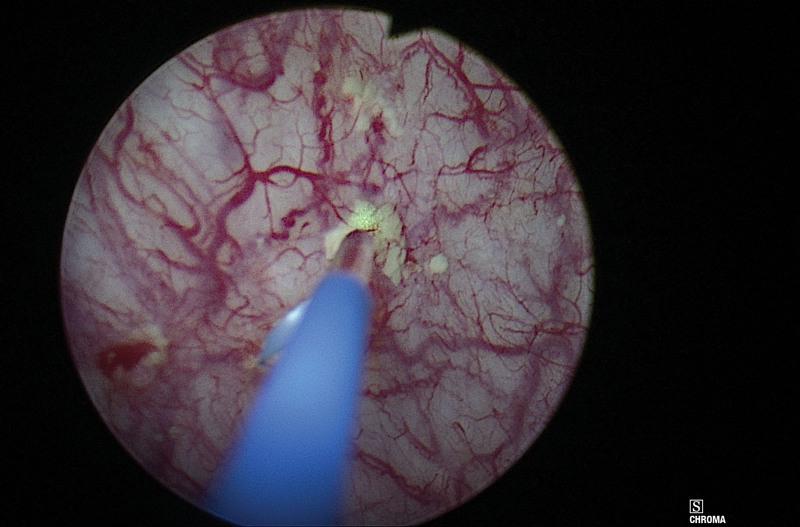
Complete removal of bladder mucosal stone with Holmium laser lithotripsy.

At the current presentation, urinalysis showed pyuria and bacteriuria. Extended spectrum beta-lactamase *Escherichia coli* (*E. coli*) was isolated from urine culture. During cystoscopy, among all quadrants of the bladder wall, there were white and flaky submucosal calcifications, and some caused mucosal breaks and stone formation (Fig. 2). Ertapenem was administered for three days; however, early *E. coli* recurrence with symptomatic infection was observed. Repeated cystoscopy revealed stationary bladder calcification. The mucosal part of the stones was removed completely (Fig. 3), and stone analysis showed 98% calcium phosphate and 2% calcium oxalate. The patient’s lower urinary tract symptoms improved greatly, and no newly formed calcifications were found at serial follow-up.

## 3. Discussion

Bladder calcification is a rare condition that is visible through either cystoscopy or radiographic imaging. The currently reported etiologies include urea-splitting bacterial infection, schistosomiasis, tuberculous infection, cancer, and chronic inflammation.^[1–3]^ CU is one of the most common causative pathogens of urea-splitting bacterial infections, and diffuse bladder mucosal calcification, also called encrusted cystitis can be found on cystoscopy.^[4,5]^

Calcification can be seen over the submucosal layer due to chronic inflammation of the bladder with calcium deposition. Histopathologically, osteoclacin can be detected in endothelial cells, fibroblasts and mononuclear cells during acute inflammation of the bladder wall, which becomes undetectable after treatment.^[6]^

Covid-19 related viral cystitis was first described by Mumm et al.^[7]^ Increased urinary frequency and nocturia were observed in patients diagnosed with Covid-19. De novo urinary tract symptoms may contribute to elevated proinflammatory cytokine levels in the urine.^[8]^ From a cohort described early in the Covid-19 pandemic, 14% of patients had severe disease, and 5% of patients were critically ill and developed organ failure.^[9]^ Cytokine storm plays a critical role in life-threatening inflammatory processes by elevating the levels of circulating cytokines.^[10]^ Inflammation related calcification of the coronary artery, bronchioles and brain has been reported in patients with symptomatic Covid-19.^[4,11,12]^ The severity of coronary artery calcification has been proposed as a predictor of adverse clinical outcomes in patients with Covid-19,^[13]^ although the actual association between an acute proinflammatory state and tissue calcification has not been confirmed biomedically.

For urea-splitting bacterial infections, antimicrobial therapy, acidification of urine, and complete removal of encrustation are the mainstays of treatment. Bladder instillation of hyaluronic acid, dimethyl sulfoxide, and submucosal Botox injection can also help treat CU- related calcification successfully.^[14,15]^ For inflammation related submucosal calcification, removal of calcification may help alleviate urinary tract symptoms.

Our interpretation that bladder calcifications may be related to Covid-19 was based on the following clinical findings. First, the patient had no history of urolithiasis and presented with de novo urinary frequency and nocturia after severe Covid-19 infection. Moreover, the urine was neutral, and only non-urease-producing pathogens were identified from repeat urine cultures throughout the course (Candida species during Covid-19 hospitalization and *E. coli* after recovery). Third, no CU aimed antibiotics were administered during the treatment, and no progression of bladder wall encrustation was noted in two cystoscopy exams at one month interval.

## 4. Conclusion

We report the case of a 68-year-old man with bladder submucosal calcification who recovered from severe Covid-19. Bladder calcification may be a urinary tract sequela of Covid-19, and more biomedical studies are warranted to confirm our findings.

## Acknowledgments

We would like to acknowledge the support of the Far Eastern Memorial Hospital. We would also like to acknowledge the contributions of our colleagues.

## Author contributions

P.Y. Cheng contributed to the conception and design, acquisition of data, and major contributor in writing of the manuscript. All authors contributed to material support.
